# The critical role of primary care providers in addressing suicide

**DOI:** 10.1007/s00406-024-01892-y

**Published:** 2024-09-09

**Authors:** Karoline Lukaschek, Puya Younesi, Carolin Haas

**Affiliations:** 1 Institute of General Practice and Family Medicine, LMU University Hospital, LMU Munich, Munich, Germany; 2 Graduate Program “POKAL - Predictors and Outcomes in Primary Care Depression Care” (DFG-GrK 2621), Munich, Germany

Suicide remains one of the most pressing public health crises, claiming over 700,000 lives annually worldwide. As stated by the WHO, suicide is a leading cause of premature mortality, and has profound impacts on families, communities, and healthcare system. Addressing this complex issue requires a multifaceted approach, with primary care settings playing a pivotal role in early detection, intervention, and prevention.

Primary care providers (PCPs) are often the first point of contact for individuals experiencing mental health issues, including those with suicidal ideation. With their unique position in the healthcare continuum, PCPs are ideally situated to identify and manage patients at risk. The integration of mental health care into primary care settings is not merely advantageous but essential. However, this integration faces significant challenges, including time constraints, lack of specialized training, and the stigmatization of mental health disorders [1]. Overcoming these challenges requires a multifaceted approach. Enhanced training programs for PCPs in mental health care, increased allocation of time for mental health consultations, and efforts to reduce stigma through education and awareness campaigns are essential.

Depression and suicidality often coexist with a variety of chronic medical conditions such as diabetes, cardiovascular diseases, and chronic obstructive pulmonary disease (COPD). The intersection of physical and mental health issues necessitates a holistic approach to patient care. PCPs must be adept at managing these comorbidities, recognizing how physical health impacts mental well-being and vice versa. Effective management of suicidality in primary care also involves understanding medication interactions and potential side effects that could exacerbate mental health conditions. Collaborative care models, which involve coordinated efforts among PCPs, mental health specialists, and other healthcare providers, are crucial in managing complex cases and providing comprehensive care [2]. These models ensure a comprehensive approach to care, addressing the complex interplay between physical and mental health and ultimately improving patient outcomes.

One of the primary obstacles in effectively managing suicidality in primary care is the limited mental health training among general practitioners (GPs). Programmes like the DFG (Deutsche Forschungsgemeinschaft, German research Foundation)-funded POKAL research training group (DFG-GRK 2621 POKAL) aim to bridge this gap by providing specialized training to GPs, enhancing their ability to recognize and treat depression and suicidality early. The training focuses on improving diagnostic accuracy, understanding comorbidities, and implementing evidence-based interventions [3]. Within POKAL, a project focussed on suicide assessment in primary care developed and evaluated of practical tools, the SuPr-10 questionnaire [4], which offers PCPs a reliable method for assessing suicide risk. It is user-friendly and applicable within the time constraints of a typical primary care visit. Ensuring that PCPs are equipped with the right tools and training can significantly enhance early detection and intervention efforts. Additionally, we conducted a systematic review in order to investigate interventions aiming at suicidal patients in a primary care setting. As a result, it can be concluded that although there are promising approaches, there is currently no satisfactory intervention available for general practitioners. This indicates a significant need for further research in this area.

While individual training and tools are vital, systemic changes in healthcare are also necessary to address suicidality effectively. This includes policy changes that prioritize mental health integration into primary care, increased funding for mental health resources, and public health campaigns to reduce stigma associated with mental health issues [5].

Furthermore, primary care systems must be designed to support the mental health needs of diverse populations, considering factors such as socio-economic status, cultural backgrounds, and access to healthcare services. Telehealth and digital health tools can also play a significant role in reaching underserved populations and providing timely mental health support [6].

The role of primary care in addressing suicidality cannot be overstated. By enhancing the training and resources available to PCPs, fostering collaborative care models, and implementing systemic changes, we can significantly improve the early detection and management of suicide risk. The stakes are high, but with concerted efforts, primary care can serve as a critical front line in the fight against suicide, ultimately saving lives and improving the quality of care for individuals at risk.

This editorial underscores the importance of ongoing research, policy development, and the implementation of effective strategies to empower primary care providers in their vital role. The integration of mental health into primary care is not just a strategic priority; it is a moral imperative to address one of the most urgent health crises of our time.

The contributions in this issue highlight the importance of identifying of risk factors for suicidal ideation and offer valuable insights for clinicians to develop targeted interventions and preventive strategies. Zhou and colleagues [7] investigated the prevalence and clinical correlates of suicidal ideation and its relationship with alexithymia, Wu and colleagues [8] presented a study exploring the sex differences in the association between suicidal ideation and neurocognitive function in Chinese patients with schizophrenia. A study by Shang and colleagues [9] highlights the significant prevalence of recent and previous suicide attempts among young Chinese Han outpatients with Psychotic Major Depressive Disorder. Turiaco and colleagues [10] shed light on the genetic background of suicidal ideation. They performed a GWAS analysis, polygenic risk score associated with a random forest analysis and a molecular pathway analysis to identify the genetic contribution to suicidal ideation. They did not find a GWAS positive result, but the molecular pathway analysis indicated a possible role of microglia and neurodevelopment in suicidal ideation.
